# A systematic review of hemorrhage and vascular injuries in civilian public mass shootings

**DOI:** 10.1186/s13049-023-01093-x

**Published:** 2023-06-19

**Authors:** Karolina Nyberger, Lovisa Strömmer, Carl-Magnus Wahlgren

**Affiliations:** 1 Department of Molecular Medicine and Surgery, Karolinska Institute, 171 76 Stockholm, Sweden; 2 Department of Vascular Surgery, Karolinska University Hospital, Stockholm, Sweden; 3 Division of Surgery, Department of Clinical Science Intervention and Technology (CLINTEC), Karolinska Institute, Stockholm, Sweden

**Keywords:** Civilian public mass shooting, Firearm injuries, Hemorrhage, Vascular injuries

## Abstract

**Background:**

Civilian public mass shootings (CPMSs) are a major public health issue and in recent years several events have occurred worldwide. The aim of this systematic review was to characterize injuries and mortality after CPMSs focusing on in-hospital management of hemorrhage and vascular injuries.

**Method:**

A systematic review of all published literature was undertaken in Medline, Embase and Web of Science January 1st, 1968, to February 22nd, 2021, according to the PRISMA guidelines. Literature was eligible for inclusion if the CPMS included three or more people shot, injured or killed, had vascular injuries or hemorrhage.

**Results:**

The search identified 2884 studies; 34 were eligible for inclusion in the analysis. There were 2039 wounded in 45 CPMS events. The dominating anatomic injury location per event was the extremity followed by abdomen and chest. The median number of operations and operated patients per event was 22 (5–101) and 10.5 (4–138), respectively. A total of 899 deaths were reported with a median mortality rate of 36.1% per event (15.9–71.4%) Thirty-eight percent (13/34) of all studies reported on vascular injuries. Vascular injuries ranged from 8 to 29%; extremity vascular injury the most frequent. Specific vascular injuries included thoracic aorta 18% (42/232), carotid arteries 6% (14/232), and abdominal aorta 5% (12/232). Vascular injuries were involved in 8.3%-10% of all deaths.

**Conclusion:**

This systematic review showed an overall high mortality after CPMS with injuries mainly located to the extremities, thorax and abdomen. About one quarter of deaths was related to hemorrhage involving central large vessel injuries. Further understanding of these injuries, and structured and uniform reporting of injuries and treatment protocols may help improve evaluation and management in the future.

*Level of Evidence* Systematic review and meta-analysis, level III.

**Supplementary Information:**

The online version contains supplementary material available at 10.1186/s13049-023-01093-x.

## Background

A civilian public mass shooting (CPMS) is an incident involving several people affected by gun violence. There is no widely accepted definition of the term mass shooting, however it is generally agreed that a mass shooting event is when three or more people are shot, injured or killed, not including the shooter [1]. CPMSs have an upward trend and in recent years, several events have occurred worldwide (e.g., Paris 2015, Las Vegas 2017, and Christchurch 2019) [2, 3]. Firearm violence is a serious public health issue globally and sufficient resources and treatment of multiple firearm injuries represent a challenge to the healthcare systems [4].

Hemorrhage remains the most preventable cause of death after firearm injuries [5, 6]. It is suggested that few survivors are severely injured after CPMSs since most of the wounded die at the scene [2]. Vascular injuries and associated hemorrhage have been shown to be particularly lethal in mass shootings where early recognition and prompt management are essential to improve survival [7]. After CPMSs, the assessment of severely injured and hemodynamically compromised patients represent a great challenge due to the large number of patients which may further increase mortality [8]. Considering the threat of domestic terrorism and a rise in gun violence, increased knowledge of hospital management and related outcomes after CPMSs would be of benefit to hospitals that strive to improve preparedness of future events. The primary aim of this systematic review was to characterize injury locations, in-hospital management of hemorrhage, vascular injuries and mortality after CPMSs.

## Methods

### Protocol and registration

The review was conducted between February 2021 and June 2021 according to the PRISMA guidelines [9]. The systematic review was registered in The International Prospective Register of Systematic Reviews (PROSPERO 2021: CRD42021275710).

### Eligibility criteria

**Fig. 1:**
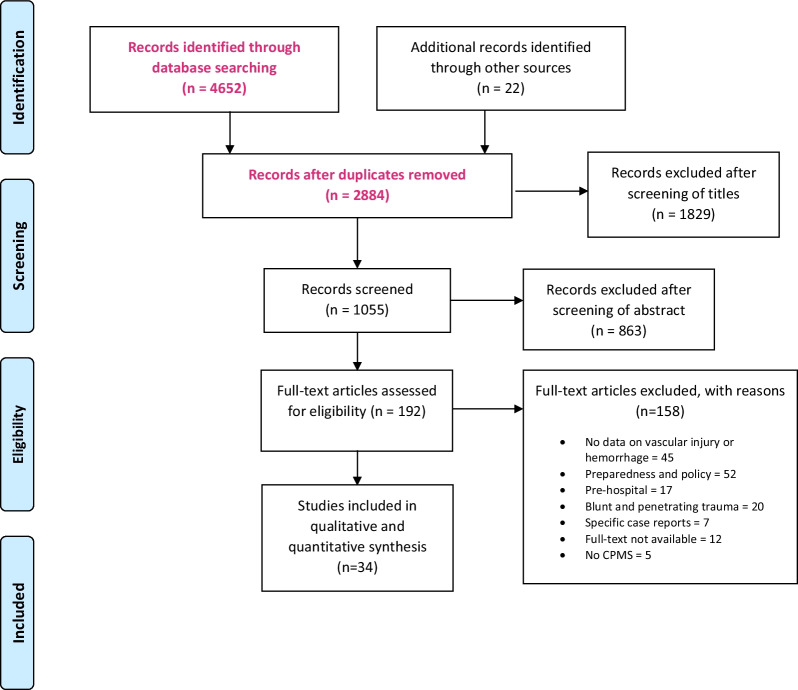
Prisma flow diagram

A systematic literature review was performed on all published scientific literature and grey literature on mass-shootings (≥ 3 people shot), vascular injuries or hemorrhage (Fig. 1).

### Information sources

Searches were applied in Medline, Embase and Web of Science of all published literature from January 1st, 1968, to February 22nd, 2021. Included in this study were all articles in the English, German, and French languages.

### Search strategy

The search aimed to retrieve all publications relating to vascular injuries and hemorrhage, after CPMSs. The medical subject heading terms were combined with non-indexed, relevant search words to identify papers on mass shootings using specific free-text phrases. In addition to medical subject heading terms for CPMSs, terms for terror attacks with firearms were also used in order to increase sensitivity. Terrorism or a terror attack was defined as premeditated, politically motivated violence perpetrated against non-combatant targets by subnational groups or clandestine agents [10]. No terms for vascular injuries or in-hospital management were used to narrow the search down. Relevant free-text terms were used in combination with controlled vocabulary where applicable. A detailed description of the search strategy, sources, and terms used are detailed in the full search strategy for Medline, Embase and Web of Science, and found in the Additional file 1: Appendix 1. A more detailed search was conducted using the Luxembourg Definition for grey literature in order to find additional material not retrieved by the initial search. Grey literature was defined as literature not controlled by commercial publishers and where publishing was not the main purpose. It included literature in print and electronic formats from government, academics, business and industry [11]. The reference list and citations from all included papers were checked for additional material not found on the original search.

### Selection Criteria

For the inclusion criteria, studies must contain data on injury locations, in-hospital management of hemorrhage (e.g. operative resources and blood transfusion requirements), vascular injuries and mortality after CPMSs as defined in the background, in English, German or French, published between January 1st, 1968, to February 22nd, 2021. Studies were excluded if they did not discuss mass casualty incidents (MCIs) due to penetrating trauma after firearm injuries with three or more people having been shot, injured or killed nor discussed data relevant to the primary aim. CPMSs including injuries, due to both explosives and firearms, were excluded if penetrating trauma due to firearm injuries was not reported separately in the study.

### Data collection and screening for eligibility

One author, (KN), screened the titles and abstracts of identified literature. Literature clearly not complying with the inclusion criteria was excluded. Abstracts deemed potentially eligible for inclusion was assessed by a second author (LS or CMW). All full-text articles were assessed for eligibility and inclusion was subject to consensus with all authors (KN, LS, and CMW).

### Primary outcomes

The primary outcomes were injury locations, in-hospital management of hemorrhage, vascular injuries and mortality after CPMSs.

### Risk of bias and quality appraisal

To assess risk of bias in the included articles, all authors agreed on exclusion of studies not in compliance with the inclusion criteria. Improper design, reporting or analysis, missing information or studies with discrepancies in reporting were therefore not eligible for inclusion. Quality was appraised by using a predefined checklist of questions depicting internal and external validity available in Additional file 1: Appendix 2 and evaluated According to the Oxford UK CEBM Levels of Evidence (www.cebm.net).

### Synthesis of results and statistical analysis

Data from all eligible articles were extracted by using a custom abstraction tool created in Microsoft Excel Version 2304 (2021 Microsoft 365, Microsoft Corporation, USA) focused on identifying common themes in the studies after CPMSs. The abstraction tool collected data under the subheadings; General information and demographics, injury locations, in-hospital management of hemorrhage (e.g. operative resources and blood transfusion requirements), vascular injuries and mortality after CPMSs. The data was summarized and presented with descriptive statistics median (min–max).

## Results

### Identification

The search identified 2884 studies; 1055 studies were included after screening of titles and abstracts. After full text reading, 34 studies were eligible for inclusion in the analysis (Fig. 1).

### Quality appraisal

Included studies comprised of case-reports (n = 19), original research papers (n = 9), commentaries (n = 4), and review articles (n = 2). The literature was of evidence levels 5 (n = 20), 4 (n = 10), and 3b (n = 4). The majority of studies was from the US (55.8%, 19/34) with additions from France (11.8%, 4/34), Norway (11.8%, 4/34), New Zealand (5.8%, 2/34) and five other countries (14.7%, 5/34); East Timor, India, Kenya, The United Kingdom and Turkey.

### General information and demographics

**Fig. 2:**
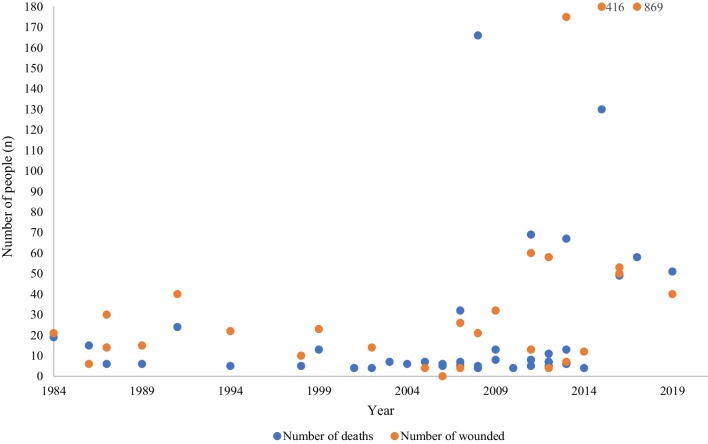
Deaths (*n* = 899) and wounded (*n* = 2039) in 45 civilian public mass shootings. Two outliers are presented with the values in the figure

Studies describing CPMSs from 45 separate events between 1984 and 2019 were included, with overlapping data in 26 CPMSs (Table 1) [2, 4, 12–43]. From the 45 separate CPMS events, the total number of people was 2039, the median number of persons per event was 36 (9–927) (Fig. 2). The median age was 31.4 years (18–43) [2, 14, 19, 22, 28, 32, 36, 39, 43]. The gender distribution; 73.9% (311/421) of all patients were men and 26.1% (110/421) women [14, 22, 28, 32, 36, 39, 43].

**Table 1:** Civilian public mass shootings between 1984 and 2019

Civilian public mass shooting (CPMS)	Year of shooting	Official deaths (n)	Official wounded	Mortality (%)
San Diego [4, 12]	1984	19	21	47.5
Edmond [4, 12]	1986	15	6	71.4
Hungerford [13]	1987	14	30	31.8
Palm Bay [14]	1987	6	14	30.0
Louisville [15]	1989	6	15	28.6
Killeen [16]	1991	24	40	37.5
Fairchild [17]	1994	5	22	18.5
Jonesboro [12]	1998	5	10	33.3
Jeffersson [4, 12, 18]	1999	13	23	36.1
Melrose Park [12, 18]	2001	4	–	–
Dili [19]	2002	–	14	–
South Bend [12, 18]	2002	4	–	–
Chicago [12, 18]	2003	7	–	–
Sawyer Country [12, 18]	2004	6	–	–
Brookfield [4]	2005	7	4	63.6
Goleta [4]	2006	6	–	–
Lancaster [12, 18]	2006	5	–	–
Colorado Springs [12, 18]	2007	5	–	–
Crandon [12]	2007	7	–	–
Omaha [4]	2007	5	4	55.6
Virginia [4, 20, 21]	2007	32	26	55.2
Dekalb [12, 18]	2008	4	–	–
Illinois [4]	2008	5	21	19.2
Mumbai [22]	2008	166	–	–
Carthage [12, 18]	2009	8	–	–
Fort Hood [23, 24]	2009	13	32	28.9
Hialeah [12, 18]	2010	4	–	–
Seal Beach [12, 18]	2011	8	–	–
Tucson [4, 12, 18]	2011	5	13	27.8
Utöya [25–28]	2011	69	60	53.5
Copley Township [12, 18]	2011	8	–	–
Aurora [4, 29]	2012	11	58	15.9
Oak Creek [4, 12, 18]	2012	7	4	63.6
Oakland [12, 18]	2012	7	–	–
Seattle [12]	2012	6	–	–
Nairobi [30]	2013	67	175	27.7
Santa Monica [12, 18]	2013	6	–	–
Seattle [18]	2013	5	–	–
Washington [4]	2013	13	7	65.0
Fort Hood [31]	2014	4	12	25.0
Paris [32–35]	2015	130	416	23.8
Istanbul [36]	2016	–	50	–
Orlando [12, 18, 37–41]	2016	49	53	48.0
Las Vegas [2, 12, 18, 41, 42]	2017	58	869	6.3
Christchurch [41, 43]	2019	51	40	56.0
Total		899	2039	

*Full list of references for each event included in Appendix 2

### Anatomical injury location

The dominating anatomic injury location per event was the extremity followed by abdomen and chest (Table 2) [2, 13, 17, 19, 23–31, 34, 36, 37, 39, 43].

**Table 2:** Anatomical injury location in civilian public mass shootings

CPMS	Head (n)	Face (n)	Neck (n)	Thorax (n)	Abdomen (n)	Spine (n)	Extremity (n)	Unspecified (n)	Multiple injuries (n)	Total number of GSW reported in study (n)	Total number of patients in CPMS (n)
Hungerford [13]					1		1			14	44
Palm Bay [14]	1		1	4	4	1	5	3	5	13	20
Louisville [15]	1			3	6	2	7	1	10	15	21
Killeen [16]				1	3		3			7	64
Fairchild [17]				1	1						27
Dili [19]				3	5		9	7	4	14	14
Fort Hood [23, 24]		1				1	2			12	45
	1			2	2		1		1	17	–
Utöya [25–28]	10*	10*		5	5		12		9	15	129
	7	7	7	11	4		10			21	–
	2			8			10			35	–
	20	13	20	32	23		30			21	–
Aurora [29]	4	1	1	3	3		6		4	23	69
Nairobi [30]	1	2		4	4	2	9			65	242
Fort Hood [31]			2	4	5		4	2	4	13	16
Paris [34]				11	10		1		2	20	546
Orlando [37, 39]	1			3	1						102
	2	2	2	10	11		26			34**	–
Istanbul [36]	5	4	5	2	2		27			50	50
Las Vegas [2]	7			17	17		34			71	927
Christchurch [43]	1			21	21			15		37	91
Total	53	30	38	145	128	6	197	28	39	497	2407

*Data reported as 10 head and neck injuries

**Data reported on 34 patients admitted of a total of 53 patients initially

### Vascular injuries and management

Thirty-eight percent (13/34) of all studies reported vascular injuries [12, 15, 17–19, 24, 32, 36, 38–40, 42]. Patients with reported vascular injuries after CPMSs ranged from 8 to 29% (Table 3). Vascular procedures, in general, included direct arterial and/or venous repair [15, 21]. There was no specific data on more advanced vascular reconstructions [15, 19, 21, 32, 39].

**Table 3:** Vascular injuries and vascular procedures in civilian public mass shootings

CPMS(s)	Vascular injuries, n (%)	Vascular injury location	Vascular procedures
Multiple CPMSs [2]	12 (6)		3.1% received tourniquet (6/191), no reported arterial injury. 1.6% underwent angiography within the first 12 h (3/191)
Louisville [15]	3 (20)	Popliteal artery; femoral vessels	Repair popliteal artery, venoraphy
Fairchild [17]	–	Vena cava; aorta	
Dili [19]	4 (29)	Mesenteric vessels; iliac vein	Peripheral limb wound exploration and control of haemorrhage, packing
Virginia [21]	–		One arterial repair
Fort Hood [23]	–	Subclavian artery; femoral artery	
Istanbul [36]	12 (8)	Femoral artery; upper extremity artery	
Paris [32] (10)	17	1.7% embolizations (5/286)	
Orlando [38, 39]	–	Tourniquet used to control bleeding temporarily 3.4% vascular procedures (3/87)	
Total	48		

### In-hospital management

The number of patients that were transferred immediately to the operating room (OR) was 20% in Louisville 1989 (3/15; one admitting hospital), 27% in Utöya 2011 (4/15; one admitting hospital) and 63% in Paris 2015 (181/286; 18 admitting hospitals). [15, 25, 32] Thirty-nine percent (28/71) of admitted patients were operated on within 12 h after the CPMS in Las Vegas 2017 [2]. In Orlando 2016, 82% (28/34) of patients and in Nairobi 2013, 63% (41/65) of all admitted patients were operated on within 24 h. [30, 39] The total number of operations (n = 392) and the number of operated patients (n = 97) were reported from eight events (Table 4). [15, 19, 20, 24, 26–28, 30, 33, 38, 39] The median number of operated patients and operations per CPMS was 10.5 (5–34) and 22 (5–101), respectively [15, 19, 20, 24, 26–28, 30, 33, 38, 39]. From studies with complete reports of injured and operated patients, 47.1% (97/206) of all hospitalized patients required a surgical procedure [15, 24, 27, 28, 30, 33]. Additional data on the most common surgical procedures, and blood transfusion requirements are presented in Additional file 1: Tables S1 and S2.

**Table 4:** Operations in civilian public mass shootings

CPMS	Operations in total (n)	Number of operated patients (n)	Hemorrhagic shock/hemothorax (n)	Total number of GSW reported in study (n)	Total number of patients in CPMS (n)
Louisville [15]	–	12	2	15	21
Dili [19]	14	–	3	14	14
Virginia [20]	10	–		17	58
Fort Hood [24]	5	5	1	17	45
Utöya [26–28]	101 10 9	– 7 9	4 4	21 35 21	129 – –
Nairobi [30]	30	30		65	242
Paris [33]	34	34	7	53	546
Orlando [37–39]			Yes		102
	86	–		53	–
	93	–		34*	–
Total	392	97	21	345	1157

*Data reported on 34 patients admitted of a total of 53 patients initially

### Mortality

From 45 mass casualty shooting events, the total number of official deaths was 899 with a median mortality rate per event of 36.1% (15.1–71.4%; 7 patients per event, 4–166) (Table 1 and Fig. 2). There were 769 autopsies after 33 separate mass shootings, including both pre- and in-hospital deaths [4, 12, 18, 22, 35, 40]. However, three of these studies included the same events (Additional file 1: Table S3). In-hospital mortality is presented in Additional file 1: Table S4. The mortality after emergency resuscitative thoracotomy was 100% (5/5) [31, 37].

Vascular injuries were involved in 8.3–10% of all deaths [18, 40]. In Orlando, with 102 wounded (53 survivors; 49 mortalities), the American Association for the Surgery of Trauma (AAST) Organ Injury Grade could be applied to 39 non-survivors and 16 survivors [39]. Non-survivors were more likely to have thoracic vascular injuries compared to survivors (12/39 versus 0/16; *P* = 0.01). Abdominal vascular injuries were present in 25% (4/16) of survivors and in 13% (5/39) of non-survivors, though with a higher organ grade. Peripheral vascular injuries seemed to be rather similar in both groups (13% survivors, 10% non-survivors, same organ grade) [39]. The most common distribution of vascular injuries after autopsy review was the thoracic aorta 18% (42/232), followed by carotid artery 6% (14/232), abdominal aorta 5% (12/232), subclavian artery 3% (7/232), inferior vena cava 2% (5/232), and superior vena cava 2% (5/232) [12]. Additionally, a multidisciplinary peer review of 19 US CPMSs showed that 15% (32/213) of all patients were deemed to have potentially preventable injuries with 31.3% (10/32) of those being intra-abdominal bleeding, 9% (3/32) vascular neck injuries and 6% (2/32) extremity injuries [18].

## Discussion

This systematic review identified 34 studies with 45 events presenting the in-hospital medical response to mass shootings of 2039 people with an overall median mortality of 36%. Injuries were mainly located to the extremities, thorax and abdomen with the dominating causes of death from thoracic and head injuries. The presence of vascular injuries ranged from 8 to 29% and almost one fourth of all deaths were related to hemorrhage where thoracic vascular injuries seemed to be the most lethal.

About one third of all patients had injuries to the extremities, which was the dominating anatomic region for firearm injuries in both CPMSs as well as in regular firearm violence [44]. The reported frequency of vascular procedures was relatively low. The anatomic vascular locations varied from extremity vessels, including femoral, popliteal, and subclavian arteries, to abdominal vessels involving aorta, vena cava, and mesenteric vessels and primary vascular repair was the dominating vascular procedure, but detailed data was lacking.

Only three studies reported the number of patients admitted directly to the OR ranging from 20 to 63% [15, 25, 32]. This most likely included patients with hemodynamic instability after injuries to the torso or extremities but also patients with peritonitis and evisceration after abdominal gunshot wounds (GSWs) [45]. Up to 82% of patients underwent surgery within 24 h, which supports the notion that most CPMS patients will need some sort of surgical intervention [39]. Blood transfusions had a large individual variation but the need for blood products was high. In Christchurch 2019, most of the blood transfusions was given to a small number of patients where approximately 10% of the patients received massive transfusion [43].

The overall median mortality in this systematic review was high but the reported in-hospital mortality was 7.4% with 38% of these wounded being dead on arrival, which implies that most patients still die at the scene of the CPMS. The most common fatal injury location was the thorax based on autopsy protocols and was deemed by multidisciplinary peer review committees to be the most common cause of potentially preventable death [12]. Gunshot wounds to the head, which is fatal approximately 90% of the time and with many patients dying before arriving to the hospital, was the second most common fatal injury location [46]. The dominating fatal vascular injuries were the thoracic and abdominal aorta and the carotid artery [12, 18, 39, 40]. These injuries are challenging to manage because of their devastating nature associated with high mortality. Damage-control resuscitation and surgical techniques with immediate bleeding control may improve in-hospital outcome after these vascular injuries [45]. The role of adjunct endovascular techniques for bleeding control in the mass casualty situation need to be further defined [47].

### Limitations

This systematic review illustrates the widespread heterogeneity in outcome measures across studies and therein its limitations due to missing data for specific variables that were not reported. Changes in standards and quality of care between countries and over time may have contributed to heterogenous data. Furthermore, there is a limitation in missing data for specific variables which were not reported in all studies. Studies were excluded if they did not report data separately for specific CPMSs. Articles with overlapping data were specified and carefully scrutinized to extract data for each specific event. Sparse vascular injury data made it difficult to draw more detailed conclusions concerning the management. The number of people included to define civilian public mass shootings is an important topic of discussion since there is no widely accepted definition. We used a broad definition requiring at least three people, either injured or killed, in an attempt to capture the full impact of these mass-shooting events. The Federal Bureau of Investigation (FBI) uses the classification three or more killed to classify a mass killing [1]. Others have defined a mass shooting whenever four or more people are shot, injured, or killed [47]. A consensus definition may help inclusion and comparison of CPMS studies. The lack of structured reporting after CPMSs contributes to difficulties in framing major conclusions regarding management of CPMSs. Further analysis of clinical data, and mortality due to hemorrhage and vascular injuries as well as uniform reporting of injuries and treatment protocols may help improve future evaluation, possible preventable measures and areas of improvement in the management of wounded CPMSs.

## Conclusion

This systematic review showed an overall high mortality after CPMSs with injuries mainly located to the extremities, thorax and abdomen. About one quarter of deaths were related to hemorrhage involving central large vessel injuries. Standardized reporting of injuries and management protocols may help improve future evaluation after CPMS.

### Supplementary Information

**Additional file 1**. **Table S1**. Surgical procedures in civilian public mass shootings. **Table S2**. Blood transfusion requirements in civilian public mass shootings. **Table S3**. Autopsies of prehospital and hospital deaths after civilian public mass shootings. **Table S4**. In-hospital mortality after civilian public mass shootings.

## Data Availability

All manuscripts where data have been generated or analysed and the full search strategy are included in this published article and in the appendix.

## Medline search strategy

**Table:**

Interface: Ovid MEDLINE(R) and Epub Ahead of Print, In-Process & Other Non-Indexed Citations and Daily Date of Search: 22 February 2021 Number of hits: 1153 Comment: In Ovid, two or more words are automatically searched as phrases; i.e. no quotation marks are needed	Field labels exp/ = exploded MeSH term / = non exploded MeSH term ti,ab,kf. = title, abstract and author keywords adjx = within x words, regardless of order * = truncation of word for alternate endings

#	Searches	Results
1	Active killer*.ti,ab,kf	26
2	(active adj (shooter* or shooting*)).ti,ab,kf	149
3	Campus shoot*.ti,ab,kf	19
4	(civilian* adj2 shoot*).ti,ab,kf	14
5	(mass adj2 (shooter* or shooting*)).ti,ab,kf	277
6	(public* adj2 shoot*).ti,ab,kf	29
7	School shoot*.ti,ab,kf	167
8	(spree adj1 (kill* or murder*)).ti,ab,kf	12
9	1 or 2 or 3 or 4 or 5 or 6 or 7 or 8	616
10	exp Terrorism/	12,906
11	Mass Casualty Incidents/	2177
12	terror*.ti,ab,kf	8742
13	Mass casualt*.ti,ab,kf	2488
14	(attack* adj3 (civilian* or mass or public)).ti,ab,kf	410
15	disaster*.ti,ab,kf	27,585
16	(major adj2 (incident* or event* or accident*)).ti,ab,kf	17,545
17	Mass fatalit*.ti,ab,kf	126
18	Mass kill*.ti,ab,kf	89
19	Mass murder*.ti,ab,kf	162
20	Massacre*.ti,ab,kf	229
21	10 or 11 or 12 or 13 or 14 or 15 or 16 or 17 or 18 or 19 or 20	61,290
22	Firearms/	5388
23	Wounds, Gunshot/	15,548
24	Gun violence/	242
25	(firearm* or gun or guns or gunshot* or handgun or pistol* or revolver* or rifle* or shooter* or shooting* or shotgun*).ti,ab,kf	37,262
26	22 or 23 or 24 or 25	44,536
27	21 and 26	803
28	9 or 27	1153

## Embase search strategy

**Table:**

Interface: embase.com Date of Search: 22 February 2021 Number of hits: 1419 Comment: Emtree is the controlled vocabulary in Embase	Field labels /exp = exploded Emtree term /de = non exploded Emtree term ti,ab,kw = title, abstract and author keywords NEAR/x = within x words, regardless of order * = truncation of word for alternate endings

No	Query	Results
#1	'Active killer*':ti,ab,kw	28
#2	(active NEAR/1 (shooter* OR shooting*)):ti,ab,kw	162
#3	'Campus shoot*':ti,ab,kw	18
#4	(civilian* NEAR/2 shoot*):ti,ab,kw	17
#5	(mass NEAR/2 (shooter* OR shooting*)):ti,ab,kw	320
#6	(public NEAR/2 shoot*):ti,ab,kw	32
#7	'School shoot*':ti,ab,kw	232
#8	(spree NEAR/1 (kill* OR murder*)):ti,ab,kw	17
#9	#1 OR #2 OR #3 OR #4 OR #5 OR #6 OR #7 OR #8	726
#10	'TERRORISM'/exp	9564
#11	'Mass disaster'/de	2785
#12	terror*:ti,ab,kw	10,677
#13	'Mass casualt*':ti,ab,kw	3051
#14	(attack* NEAR/3 (civilian* OR mass OR public)):ti,ab,kw	449
#15	Disaster*:ti,ab,kw	31,898
#16	(major NEAR/2 (incident* OR event* OR accident*)):ti,ab,kw	28,184
#17	'Mass fatalit*':ti,ab,kw	147
#18	'Mass kill*':ti,ab,kw	97
#19	'Mass murder*':ti,ab,kw	208
#20	Massacre*:ti,ab,kw	304
#21	#10 OR #11 OR #12 OR #13 OR #14 OR #15 OR #16 OR #17 OR #18 OR #19 OR #20	75,896
#22	'Firearm'/exp	6118
#23	'Gunshot injury'/de	20,015
#24	'Gun violence'/de	439
#25	(firearm* OR gun OR guns OR gunshot* OR handgun OR pistol* OR revolver* OR rifle* OR shooter* OR shooting* OR shotgun*):ti,ab,kw	44,647
#26	#22 OR #23 OR #24 OR #25	53,219
#27	#21 AND #26	988
#28	#9 OR #27	1419

## Web of science core collection search strategy

**Table:**

Interface: Clarivate Analytics Date of Search: 22 February 2021 Number of hits: 2080	Field labels TS/Topic = title, abstract, author keywords and Keywords Plus NEAR/x = within x words, regardless of order * = truncation of word for alternate endings Note: sometimes “quotation marks” are needed for single search terms to avoid automatic term mapping (lemmatization)

Set	Query	Results
#1	TS = (“active killer*”)	18
#2	TS = (“active” NEAR/1 (shooter* or shooting*))	173
#3	TS = (“campus shoot*”)	35
#4	TS = (civilian* NEAR/2 (shoot*))	40
#5	TS = (“mass” NEAR/2 (shooter* OR shooting*))	579
#6	TS = (public* NEAR/2 shoot*)	106
#7	TS = (“school shoot*”)	512
#8	TS = (“spree” NEAR/1 (kill* OR murder*))	33
#9	#8 OR #7 OR #6 OR #5 OR #4 OR #3 OR #2 OR #1	1266
#10	TS = (attack* NEAR/3 (civilian* OR “mass” OR “public”))	1720
#11	TS = (disaster*)	93,576
#12	TS = (“major” NEAR/2 (incident* or event* or accident*))	41,964
#13	TS = ”mass casualt*”	2331
#14	TS = ”mass fatalit*”	141
#15	TS = (“mass kill*”)	401
#16	TS = (“mass murder*”)	746
#17	TS = (massacre*)	3739
#18	TS = (terror*)	48,600
#19	#18 OR #17 OR #16 OR #15 OR #14 OR #13 OR #12 OR #11 OR #10	187,022
#20	TS = (firearm* or gun or guns or gunshot* or handgun or pistol* or revolver* or rifle* or shooter* or shooting* or shotgun*)	82,735
#21	#20 AND #19	1164
#22	#9 OR #21	2080

## Appendix 2. Quality appraisal and data extraction.

**Table:**

**CPMS**	**Hungerford** [13]	Palm Bay [14]	**Louisville** [15]	**Killeen** [16]	**Fairchild** [17]	**Dili** [19]	**Virginia** [20, 21]		**Mumbai** [22]	Fort Hood [23, 24]		**Utöya** [25–28]			**Aurora** [29]	
Author	Forrester et al.	Curry	Richardson et al.	Early et al.	Beyersdorf et al.	Guest et al.	Armstrong et al.	Kaplowitz et al.	Bhandarwar et al.	Shepherd et al.	Wild et al.	Young et al.	Jorgensen et al.	Waage et al.	Gaarder et al.	Koehler et al.
Year	1987	1987	1989	1991	1994	2002	2007		2008	2009		2011				2012
Article type	Casereport	Commentary	Casereport	Casereport	Casereport	Casereport	Commentary	Casereport	Original Article	Commentary	Casereport	Casereport	Original Article	Casereport	Casereport	Casereport
*Quality appraisal*																
1. Literature indexed?	✔	✔	✔		✔	✔	✔	✔	✔	✔	✔	✔	✔	✔	✔	✔
2. Authors involved in medical response?	✔	✔	?	✔		✔		?	?	✔	?	✔	?	✔	?	?
3. Reference to data sources provided?	✔		✔	✔	✔	✔	✔		✔	✔	✔	✔	✔			
4. Conflict of interest depicted?						✔		✔		✔	✔	✔				
5. Ethical approval provided?				?					✔	✔	✔	✔	✔			
6. Response clearly described?	✔	✔		✔	✔	✔		✔	✔	✔		✔	✔	✔	✔	
7. Incident clearly described?	✔	✔	✔	✔	✔			✔	✔	✔	✔	✔		✔	✔	✔
8. Indication of missing data?										?	✔			✔		
9. Other limitations discussed?	✔									✔	✔					
10. Study design clearly described?	✔							✔	✔		✔	✔		✔	✔	
*Data extraction*																
Injury location	✔	✔	✔	✔	✔	✔			✔	✔	✔	✔	✔	✔	✔	✔
In-hospital management of hemorrhage	✔	✔	✔			✔	✔	✔		✔	✔	✔	✔	✔	✔	✔
Vascular injuries and hemorrhage	✔		✔		✔	✔		✔	✔		✔		✔		✔	
Mortality	✔	✔	✔	✔		✔	✔	✔	✔		✔		✔	✔	✔	✔

**Table:**

Nairobi [30]	Fort Hood [31]	Paris [32–35]				Istanbul [36]	Orlando [37–40]				Las Vegas [2, 41]		Christchurch [43]	Multiple [2, 4, 12, 18, 41]			
Wachira et al.	Strommen et al.	Raux et al.	Borel et al.	Boddaert et al.	Tracqui et al.	Açiksari et al.	Cheatham et al.	Spruce et al.	Smith et al.	Smith et al.	Sarani et al.	Lozada et al.	Badami et al.	Smith et al.	Sarani et al.	Smith et al.	Ramsey
2013	2014	2015				2016	2016				2017		2019				
Casereport	Casereport	Original Article	Casereport	Casereport	Casereport	Original Article	Commentary	Review	Casereport	Casereport	Original Article	Original Article	Casereport	Original Article	Original Article	Original Article	Review
✔	✔	✔	✔	✔	✔	✔	✔	✔	✔	✔	✔	✔	✔	✔	✔	✔	✔
✔	?	?	?	?	✔	✔	✔		✔			?					
		✔		✔	✔	✔			✔	✔	✔	✔	✔	✔	✔	✔	✔
✔	✔	✔	✔	✔	✔	✔		✔	✔		✔	✔	✔	✔	✔	✔	✔
✔	✔	?			✔	✔			✔	✔	✔			?	✔	✔	
		✔	✔	✔	✔	✔	✔	✔	✔		✔	✔	✔				
✔	✔	✔	✔	✔		✔	✔		✔	✔	✔	✔	✔				
		✔		✔					✔	✔	✔				✔		✔
✔		✔	✔	✔	✔	✔				✔	✔			✔	✔	✔	✔
✔	✔	✔		✔		✔			✔	✔	✔	✔		✔	✔	✔	✔
✔	✔	✔		✔	✔	✔	✔		✔	✔	✔		✔	✔	✔	✔	
✔	✔	✔	✔			✔	✔	✔	✔	✔	✔	✔	✔				✔
		✔	✔		✔	✔	✔	✔	✔	✔			✔		✔	✔	✔
✔	✔	✔	✔		✔		✔		✔	✔		✔		✔	✔	✔	

## Appendix 3. Full list of included Civilian public mass shooting events and references.

**Table:**

Civilian public mass shooting	Year of shooting	Name of article	Author (s)
San Diego [4, 12]	1984	The profile of wounding in civilian public mass shooting fatalities [4]	Smith et al.
		Wounding Patterns Based on Firearm Type in Civilian Public Mass Shootings in the United States [12]	Sarani et al.
Edmond [4, 12]	1986	The profile of wounding in civilian public mass shooting fatalities [4]	Smith et al.
		Wounding Patterns Based on Firearm Type in Civilian Public Mass Shootings in the United States [12]	Sarani et al.
Hungerford [13]	1987	The Hungerford Disaster A Late Perspective of the Military Experience [13]	Forester et al.
Palm Bay [14]	1987	A disaster that could happen anywhere -the Palm Bay massacre [14]	Curry
Louisville [15]	1989	After the Shooting Stops: Follow-up on Victims of an Assault Rifle Attack [15]	Richardson et al.
Killeen [16]	1991	Darnall Army Community Hospital's response to the Killeen Massacre [16]	Early et al.
Fairchild [17]	1994	Community medical response to the Fairchild mass casualty event [17]	Beyersdorf et al.
Jonesboro [12]	1998	Wounding Patterns Based on Firearm Type in Civilian Public Mass Shootings in the United States [12]	Sarani B et al.
Jeffersson [4, 12, 18]	1999	The profile of wounding in civilian public mass shooting fatalities [4] Wounding Patterns Based on Firearm Type in Civilian Public Mass Shootings in the United States [12]	Smith et al. Sarani et al.
		Incidence and Cause of Potentially Preventable Death after Civilian Public Mass Shooting in the US [18]	Smith et al.
Melrose Park [12, 18]	2001	Wounding Patterns Based on Firearm Type in Civilian Public Mass Shootings in the United States [12] Incidence and Cause of Potentially Preventable Death after Civilian Public Mass Shooting in the US [18]	Sarani et al. Smith et al.
Dili [19]	2002	Back to basic: Managing gunshot injuries in East Timor [19]	Guest et al.
South Bend [12, 18]	2002	Wounding Patterns Based on Firearm Type in Civilian Public Mass Shootings in the United States [12] Incidence and Cause of Potentially Preventable Death after Civilian Public Mass Shooting in the US [18]	Sarani et al. Smith et al.
Chicago [12, 18]	2003	Wounding Patterns Based on Firearm Type in Civilian Public Mass Shootings in the United States [12] Incidence and Cause of Potentially Preventable Death after Civilian Public Mass Shooting in the US [18]	Sarani et al. Smith et al.
Sawyer Country [12, 18]	2004	Wounding Patterns Based on Firearm Type in Civilian Public Mass Shootings in the United States [12] Incidence and Cause of Potentially Preventable Death after Civilian Public Mass Shooting in the US [18]	Sarani et al. Smith et al.
Brookfield [4]	2005	The profile of wounding in civilian public mass shooting fatalities [4]	Smith et al.
Goleta [4]	2006	The profile of wounding in civilian public mass shooting fatalities [4]	Smith et al.
Lancaster [12, 18]	2006	Wounding Patterns Based on Firearm Type in Civilian Public Mass Shootings in the United States [12] Incidence and Cause of Potentially Preventable Death after Civilian Public Mass Shooting in the US [18]	Sarani et al. Smith et al.
Colorado Springs [12, 18]	2007	Wounding Patterns Based on Firearm Type in Civilian Public Mass Shootings in the United States [12] Incidence and Cause of Potentially Preventable Death after Civilian Public Mass Shooting in the US [18]	Sarani et al. Smith et al.
Crandon [12]	2007	Wounding Patterns Based on Firearm Type in Civilian Public Mass Shootings in the United States [12]	Sarani et al.
Omaha [4]	2007	The profile of wounding in civilian public mass shooting fatalities [4]	Smith et al.
Virginia [4, 20, 21]	2007	The profile of wounding in civilian public mass shooting fatalities [4]	Smith et al.
		Lessons from the response to the Virginia Tech shootings [20] Regional Health System Response to the Virginia Tech Mass Casualty Incident [21]	Armstrong et al. Kaplowitz et al.
Dekalb [12, 18]	2008	Wounding Patterns Based on Firearm Type in Civilian Public Mass Shootings in the United States [12] Incidence and Cause of Potentially Preventable Death after Civilian Public Mass Shooting in the US [18]	Sarani et al. Smith et al.
Illinois [4]	2008	The profile of wounding in civilian public mass shooting fatalities [4]	Smith et al.
Mumbai [22]	2008	Mortality pattern of the 26/11 Mumbai terror attacks [22]	Bhandarwar et al.
Carthage [12, 18]	2009	Wounding Patterns Based on Firearm Type in Civilian Public Mass Shootings in the United States [12] Incidence and Cause of Potentially Preventable Death after Civilian Public Mass Shooting in the US [18]	Sarani et al. Smith et al.
Fort Hood [23, 24]	2009	Are you ready? Lessons learned from the Fort Hood shooting in Texas [23] The Fort Hood Massacre: Lessons learned from a high-profile mass casualty [24]	Shepherd et al. Wild et al.
Hialeah [12, 18]	2010	Wounding Patterns Based on Firearm Type in Civilian Public Mass Shootings in the United States [12] Incidence and Cause of Potentially Preventable Death after Civilian Public Mass Shooting in the US [18]	Sarani et al. Smith et al.
Seal Beach [12, 18]	2011	Wounding Patterns Based on Firearm Type in Civilian Public Mass Shootings in the United States [12] Incidence and Cause of Potentially Preventable Death after Civilian Public Mass Shooting in the US [18]	Sarani et al. Smith et al.
Tucson [4, 12, 18]	2011	The profile of wounding in civilian public mass shooting fatalities [4]	Smith et al.
		Wounding Patterns Based on Firearm Type in Civilian Public Mass Shootings in the United States [12] Incidence and Cause of Potentially Preventable Death after Civilian Public Mass Shooting in the US [18]	Sarani et al. Smith et al.
Utöya [25–28]	2011	Radiology response in the emergency department during a mass casualty incident: a retrospective study of the two terrorist attacks on 22 July 2011 in Norway [25]	Young et al.
		Injuries caused by fragmenting rifle ammunition [26] Rural hospital mass casualty response to a terrorist shooting spree [27] The twin terrorist attacks in Norway on July 22, 2011: The trauma centre response [28]	Jorgensen et al. Waage et al. Gaarder et al.
Copley Township [12, 18]	2011	Wounding Patterns Based on Firearm Type in Civilian Public Mass Shootings in the United States [12] Incidence and Cause of Potentially Preventable Death after Civilian Public Mass Shooting in the US [18]	Sarani et al. Smith et al.
Aurora [4, 29]	2012	The profile of wounding in civilian public mass shooting fatalities [4] Surviving the dark night: The Aurora Colorado, Mass Shooting [29]	Smith et al. Koehler et al.
Oak Creek [4, 12, 18]	2012	The profile of wounding in civilian public mass shooting fatalities [4]	Smith et al.
		Wounding Patterns Based on Firearm Type in Civilian Public Mass Shootings in the United States [12] Incidence and Cause of Potentially Preventable Death after Civilian Public Mass Shooting in the US [18]	Sarani et al. Smith et al.
Oakland [12, 18]	2012	Wounding Patterns Based on Firearm Type in Civilian Public Mass Shootings in the United States [12] Incidence and Cause of Potentially Preventable Death after Civilian Public Mass Shooting in the US [18]	Sarani et al. Smith et al.
Seattle [12]	2012	Wounding Patterns Based on Firearm Type in Civilian Public Mass Shootings in the United States [12]	Sarani et al.
Nairobi [30]	2013	Westgate Shootings: An Emergency Department Approach to a Mass-casualty Incident [30]	Wachira et al.
Santa Monica [12, 18]	2013	Wounding Patterns Based on Firearm Type in Civilian Public Mass Shootings in the United States [12] Incidence and Cause of Potentially Preventable Death after Civilian Public Mass Shooting in the US [18]	Sarani et al. Smith et al.
Seattle [12]	2013	Incidence and Cause of Potentially Preventable Death after Civilian Public Mass Shooting in the US [18]	Smith et al.
Washington [4]	2013	The profile of wounding in civilian public mass shooting fatalities [4]	Smith et al.
Fort Hood [31]	2014	2014 Fort Hood, Texas, mass casualty incident: reviews and perspectives [31]	Strommen et al.
Paris [32–35]	2015	Analysis of the medical response to November 2015 Paris terrorist attacks: resource utilization according to the cause of injury [32] Feedback on terrorist attacks on November 13, 2015. Mass casualty management in trauma center [33] Surgical management of penetrating thoracic injuries during the Paris attacks on 13 November 2015 [34] An overview of forensic operations performed following the terrorist attacks on November 13, 2015, in Paris [35]	Raux et al. Borel et al. Boddaert et al. Tracqui et al.
Istanbul [36]	2016	The characteristics of the patients in mass public shootings among coup attempt in Turkey: A single-center hospital response [36]	Açiksari et al.
Orlando [12, 18, 37–41]	2016	Wounding Patterns Based on Firearm Type in Civilian Public Mass Shootings in the United States [12] Incidence and Cause of Potentially Preventable Death after Civilian Public Mass Shooting in the US [18] Orlando Regional Medical Center responds to Pulse nightclub shooting [37] Back to basics: Mass Casualty Incidents [38] Injury characteristics of the Pulse Nightclub shooting: Lessons for mass casualty incident preparation [39] Fatal Wounding Pattern and Causes of Potentially Preventable Death Following the Pulse Night Club Shooting Event [40] Blood transfusions in mass casualty events: recent trends [41]	Sarani et al. Smith et al. Cheatham et al. Spruce et al. Smith et al. Smith et al. Ramsey
Las Vegas [2, 12, 18, 41, 42]	2017	Characteristics of Survivors of Civilian Public Mass Shootings: An East Multicenter Study [2] Wounding Patterns Based on Firearm Type in Civilian Public Mass Shootings in the United States [12] Incidence and Cause of Potentially Preventable Death after Civilian Public Mass Shooting in the US8 [18] Blood transfusions in mass casualty events: recent trends [41] The Las Vegas mass shooting: An analysis of blood component administration and blood bank donations [42]	Sarani et al. Sarani et al. Smith et al. Ramsey Lozada et al.
Christchurch [41, 43]	2019	Blood transfusions in mass casualty events: recent trends [41] Analysis of transfusion therapy during March 2019 mass shooting incident in Christchurch New Zealand [43]	Ramsey Badami et al.
