# Supplementary material for: A systematic review of hemorrhage and vascular injuries in civilian public mass shootings
Source: Scand J Trauma Resusc Emerg Med. 2023 Jun 19;31:30. doi: 10.1186/s13049-023-01093-x (PMC10280979; doi:10.1186/s13049-023-01093-x)
Supplement: Supplementary file 1 — Additional file 1. Table S1. Surgical procedures in civilian public mass shootings. Table S2. Blood transfusion requirements in civilian public mass shootings. Table S3. Autopsies of prehospital and hospital deaths after civilian public mass shootings. Table S4. In-hospital mortality after civilian public mass shootings. [file 13049_2023_1093_MOESM1_ESM.docx]

**Supplementary table 1.**

| **CPMS** | **Laparotomy** | **Thoracotomy** | **Craniotomy** | **Chest drain** | **Orthopedic procedure** | **Debridement/ foreign body removal** | **Specifications** |
| --- | --- | --- | --- | --- | --- | --- | --- |
| Hungerford^13^ |  |  |  |  | 1 |  | Fasciotomy (n=1) |
| Palm Bay^14^ | 4 | 1 |  |  | 1 |  | Colon injury (n=1), bowel resection (n=2),  drainage abdominal abscess (n=1),  liver laceration (n=2), pancreas injury (n=1), lobectomy (n=1), removal C6/C7 spinous processus (n=1) |
| Louisville^15^ | 11 | 1 | 1 | 2 | 6 | 12 | Bowel resection (n=6), stoma (n=6), nephrectomy (n=1), lung resection (n=1), fasciotomy (n=1), other orthopedic procedures (n=5) |
| Dili^19^ | 3 |  |  | 3 | 2 | 14 | Bowel resection (n=1), stoma (n=1), liver laceration with repair of diaphragm (n=1), fracture manipulation (n=2) |
| Virginia^21^ | 3 |  |  | 1 |  | 4 |  |
| Fort Hood^24^ | 1 |  | 1 |  | 2 |  | Splenectomy (n=1) |
| Utöya^26,27^ | 6 | 1 | 1 | 8 | 15 | 138 | Amputation (n=5), fasciotomy (n=2), fracture treatment (n=6), external fixation (n=2) |
|  | 1 |  |  | 4 |  | 7 |  |
| Fort Hood^31^ | 1 | 2 |  |  | Yes |  |  |
| Paris^32,33^ |  |  |  | 12 |  |  |  |
|  | 7 |  |  |  |  |  |  |
| Istanbul^36^ |  |  |  |  | 30 | 76 |  |
| Orlando^37,39^ |  | 3 |  |  |  |  |  |
|  | 13 |  |  |  |  |  |  |
| **Total** | **50** | **8** | **3** | **30** | **57** | **251** | **399** |

**Supplementary table 2.**

| **CPMS** |  | Patients transfused (n) | Red blood cells (n) | Platelets (n) | Fresh frozen plasma (n) | MTP (n) | Comment(s) |
| --- | --- | --- | --- | --- | --- | --- | --- |
| Multiple^2^ |  |  |  |  |  |  | The median shock index on arrival to the trauma centre was 0.74 (IQR 0.62-0.88) |
| Dili^19^ |  | 4 |  |  |  |  | Total units transfused was 8 in 14 patients. |
| Virginia^21^ |  | 1 |  |  |  |  |  |
| Utöya^27^ |  | 6 |  |  |  |  |  |
| Paris^32^ |  | 47 |  |  |  |  |  |
| Orlando^38,39,41^ |  |  | 249 | 25 | 160 |  | 411 blood products transfused in 24 hours.  171 units of RBCs, 116 units of FFP and 25 units of platelets were transfused within the first 24 hours.  434 blood products transfused in 24 hours. |
| Las Vegas^41,42^ |  |  | 500 | 87 | 270 |  | 1.5 units of red blood cells (RBCs)/admission, 0.82 units of fresh frozen plasma (FFP)/admission and 0.24 units of platelets per admission. 857 blood products transfused in 26 hours. |
|  |  | | 278 | 45 | 151 |  | Total units transfused 499 in 519 patients. |
| Christchurch^41,43^ |  |  | 148 | 14 | 115 |  | 277 blood products transfused in 26 hours. |
|  |  | 24 | 281 | 23 | 198 | 8 | Total units transfused 583 in 72 patients. 11.1% received MT (8/72). 24 patients were treated with a total of 583 units transfused where one patient received 199 of all units transfused, two patients received 75 and 85 units, respectively, four patients 30-35 units, three patients 5-10 units, and nine patients 1-4 units. |
| **Total** |  | **82** | **1456** | **194** | **894** |  |  |

**Supplementary table 3.**

| **Autopsies**  Event(s) | Autopsies (n) | Autopsies/total deaths (%) | Fatal injury location* | |  |  | Potentially survivable injuries (%) |  |
| --- | --- | --- | --- | --- | --- | --- | --- | --- |
|  |  |  | Head (%) | Thorax (%) | Abdomen (%) | Extremity  (%) | | Comment (s) |
| Multiple CPMS^4^ | 139 | 100 | 39 | 38 | 7 | - | 7 | Head or thoracic injuries were reported as the most common causes of death according to injury locations reported as the cause of death in 77% of 139 autopsies. Thoracic injury was found to be a major cause of preventable death. |
| Multiple CPMS^12^ | 232 | - | 39 | 51 | 9 | 3 | 16 | The cause of death due to thoracic injury and head injury were 51% and 39% respectively. |
| Multiple CPMS^18^ | 213 | 40.3 | 38 | 46 | 22 |  | 15 | The cause of death due to thoracic injury and head injury were 46% and 38% respectively. |
| Mumbai^22^ | 68 | 41.0 | - | - | - | - | - | 9 patients died due to hypovolemic shock. |
| Paris^35^ | 68 | 52.7 | - | - | - | - | - | Fifty-three percent (68/128) of all deaths were autopsied after the Paris 2015 CPMS and craniocerebral injuries, extensive organ lacerations and/or massive hemorrhage were reported as common causes of death. |
| Orlando^40^ | 49 | 100 | 24 | 41 | 12 | 8 | 32 | The cause of death due to thoracic injury and head injury were 53% and 24% respectively. |
| **Total** | **769** |  |  |  |  |  |  |  |

**Supplementary table 4.**

| **CPMS** | Number of deaths at hospital (n) | Cause of death  Head | Thorax | Abdomen | Major vascular injury/ hemorrhage | Number of patients dead upon arrival (n) | Total number of GSW reported in study (n) | Total number of patients in CPMS (n) |
| --- | --- | --- | --- | --- | --- | --- | --- | --- |
| Hungerford^13^ | 3 | 1 |  |  | 2 |  | 14 | 44 |
| Palm Bay^14^ | 3 | 1 | 1 | 1 |  |  | 13 | 20 |
| Louisville^15^ | 1 |  |  |  | 1 |  | 15 | 21 |
| Killeen^16^ | 1 |  |  | 1 |  |  | 7 | 64 |
| Dili^19^ | 1 |  |  |  | 1 |  | 14 | 14 |
| Virginia^21^ | 2 |  |  |  |  | 1 | 26 | 58 |
| Fort Hood^24^ | 3 |  | 1 |  |  |  | 17 | 45 |
| Utöya^26,28^ | 1  1 | 1 |  |  |  |  | 21  21 | 129  - |
| Aurora^29^ | 4 | 2 | 1 | 1 |  |  | 23 | 69 |
| Nairobi^30^ | 1 |  |  |  |  |  | 65 | 242 |
| Fort Hood^31^ | 2 |  | 1 |  |  |  | 13 | 16 |
| Paris^32,33^ | 7 |  |  |  |  |  | 286 | 546 |
|  | 2 |  |  |  |  | 2 | 53 | - |
| Orlando^38,39^ | 9  9 |  | 1 |  |  | 5 | 34* | 102  - |
| Las Vegas^42^ | 27 |  |  |  |  | 22 | 519 | 927 |
| Christchurch^43^ | 2 |  |  |  |  |  | 37 | 91 |
| **Total** | **79** | **5** | **5** | **3** | **4** | **30** | **1178** | **2388** |

***Data reported on 34 patients admitted of a total of 53 patients initially.**
